# The Chemistry and Physiological Action of Mercury as Used in Amalgam Fillings

**Published:** 1882-04

**Authors:** E. S. Talbot


### THE CHEMISTRY AND PHYSIOLOGICAL ACTION OF MERCURY AS USED IN AMALGAM FILLINGS.

In the construction of an amalgam two changes take place, the first being the mechanical, or mixing together of the ingredients, the second the chemical, or hardening of the composition. My first experiment was to place metallic mercury in a four-ounce glass-stoppered bottle, and submit it to the gold test, by suspending a piece of gold foil in the centre of the bottle, taking care that it did not come in contact with the sides, and cementing the stopper. I placed it under different temperatures of from 20 to 1300° F. In about thirty-six hours the surface of the gold became coated with mercury, giving it a gray color. This proved the evaporation of the metal. I then mixed a quantity of the Chicago Refining Co.'s amalgam, according to their formula, three parts of mercury to eight parts of fillings, and subjected it to the same test. The reagent was not sufficiently delicate to produce any perceptible change. Wishing to bring the highest chemical skill to bear upon the experiments, I consulted Prof. Haines, of Rush Medical College, who kindly assisted me. At his suggestion, I procured two delicate reagents, the ammonio-nitrate of silver and the chloride of platinum. In preparing the ammonio-nitrate of silver I allowed thirty grains of nitrate of silver to the ounce of water, and put a small quantity of the solution in the test-tube. This was heated, and aqua ammonia added until a precipitate formed. Increasing the aqua ammonia until the precipitate cleared up, I took a quill, and, with this liquid, wrote upon white paper. After putting the substance to be tested in the bottle, the strip of paper was placed across the mouth of the bottle, and the stopper cemented. Should a vapor arise, the liquid would become black. Leaving the bottle for ten minutes, I examined it again, and found the writing in plain black coloring. The chloride of platinum produced the same results, but required more time to accomplish it. The rapidity with which the evaporation of mercury takes place depends upon the amount of heat and the surface exposed, and not upon the quantity of mercury contained in the fillings. Thus a jar containing one quart of water would evaporate the same quantity as a jar of like surface containing a gallon, the latter taking four times longer to empty.

In the following experiments, I attached a thermometer to a water bath, and heated to the temperature of the body, 98° to 100° F., to maintain an even temperature. I conducted these experiments in the dark, as the rays of light decompose the ammonio-nitrate of silver. The strips of paper containing the reagent were placed in the mouths of all the bottles, including an empty bottle, which was used in each experiment, to prove there was no mistake.

Experiment No. 2.—Three bottles were prepared. In the first was placed an amalgam filling made from Chicago Refining Company's amalgam, according to their formula. In the second was placed an amalgam filling of like size, containing five grains more of mercury. In the third bottle there was nothing. After a lapse of ten minutes I examined the bottles and found the writing on the paper across the mouth of bottles Nos. 1 and 2 was black, while there was no discoloration of paper in the third bottle.

Experiment No. 3.—A repetition of No. 2, with the exception of the reagent, chloride of platinum being substituted for the ammonio-nitrate of silver. The results were the same in both. The time required for the latter being ten hours, while but ten minutes were required for the ammonio-nitrate of silver. In conducting the remainder of the experiments the ammonio-nitrate of silver was employed, it being the more delicate reagent, consequently producing a more marked impression, and also consuming less time than chloride of platinum.

Experiment No. 4.—Two bottles were prepared. In the first bottle were placed scraps of amalgam six months old. In the second there was nothing. In ten minutes the writing on the paper was black in the first bottle and uncolored in the second.

Experiment No. 5.—Bottle No. 1 contained amalgam fillings which had remained in the teeth from two to ten years. Bottle No. 2 was empty. The results in both being the same as in experiment No. 4.

Experiment No. 6.—In bottle No. 1 I put an amalgam filling which had been in the mouth sixteen years. In bottle No. 2 there was nothing. At the end of twenty-four hours I found the paper discolored in the first, and not in the second.

Experiment No. 7.—To demonstrate that nothing in the composition of the fresh fillings could cause the discoloration I allowed some fillings to remain sealed in the bottle for twenty-four hours. At the end of that time discovered no signs of color on the paper.

Experiment No. 8.—I procured four preparations of mercurius vivus.

| | | | | | | |
|---|---|---|---|---|---|---|
| No. 1 | contained | $\frac{1}{10}$ gr. | mercury | to 1 gr. | of sugar | of milk. |
| No. 2 | " | $\frac{1}{100}$ | " | " | " | " |
| No. 3 | " | $\frac{1}{1000}$ | " | " | " | " |
| No. 4 | " | $\frac{1}{1000000}$ | " | " | " | " |

A small quantity of each of these preparations was placed in bottles marked 1, 2, 3, 4. In No. 5 there was nothing save the reagent. The effect was alike in each of the four bottles containing the mixture. Those having the greatest quantity of mercury caused the deepest color to the paper and required less time. As before, No. 5 was unaffected.

The *Dental Register*, January, 1872, has the following case of poisoning from mercury in a tooth filling: "John T. Smith died from salivation, caused from having a tooth filled with amalgam. Dr. Sprague attended the case, and afterward called Drs. Davis and Buffin, all of whom agreed that he was suffering from the effects of mercury present in the amalgam used in filling one of his teeth. The filing had salivated the unfortunate man, and, as the inside of his mouth, throat, and windpipe swelled, respiration was hindered, and finally ceased altogether. Dr. Davis made the post-mortem examination in the presence of the coroner and jury of inquest, opening the chest, taking out the lungs, and extracting the filled tooth. No signs of any other diseases were found, except that caused by the mercury, and it was made clear to the jury by the Doctor that this caused his death. The jury returned a verdict that the deceased came to his death by suffocation, caused by inflammation of the glands and infiltration of the tissues of the neck, producing closing of the trachea by pressure thereon; 'and we further believe that the above causes were brought about by the action of mercury, used in filling the second molar tooth of the right side of the lower jaw, by Dr. E. D. Keef, Marysville, Kansas.'"

A case in practice: A lady from one of the towns in Illinois came to Chicago for treatment, having been troubleed with dyspepsia and nervous debility for two years. While under the physician's care she complained constantly of a peculiar feeling and taste in her mouth. The doctor suspected the trouble might arise from a rubber plate which she had worn for four years, and advised her to consult me. Upon examination, I found a full upper plate, composed of rubber, and on the lower jaw the molars were gone, except the second and third upon the left side. In the crown of the wisdom tooth was a large amalgam filling, and also one in the crown and posterior approximal surface extending to the free margin of the gum in the second molar. These had been inserted about two years previous. I noticed that the gums and the mucous lining of the mouth and salivary glands were quite tender. There was a strong metallic taste in the mouth, and a metallic odor to the breath. She had a peculiar paralyzed sensation in the left side of the tongue, which she had experienced for two years. She also informed me that the saliva flowed so freely that at night her clothing and pillow were saturated, and estimated the loss of saliva each night to equal one pint. I suggested the removal of the amalgam fillings and rubber plate, and substitution of gold. She assented to the proposition, and as early as possible I undertook the operations. Upon removing the amalgam fillings and applying the rubber dam, the saliva flowed in streams, completely saturating several towels. After refilling the teeth, and inserting a gold plate, the unpleasant sensation in the tongue and metallic taste disappeared. At the end of two weeks the glands were greatly improved, and the soreness under the tongue (of which she had complained at her first visit) was healed.

Mercury taken into the system in small quantities, long continued, manifests itself in a variety of ways. One of the first symptoms noticeable is an increased flow of the secretions of the body—salivation being the most striking—the glands becoming inflamed and the mucous membrane tender. The gums tumefy and change in color to a dark rose tint ; the tongue is swollen ; the patient not only experiences an unpleasant metallic taste, but the breath becomes impregnated also. Sometimes extensive ulcers attack the throat, gums, and cheeks ; œdema of the glottis, with difficulty in breathing and swallowing. The digestive apparatus is involved, with loss of appetite, nausea and vomiting, and frequently pain and tenderness of the stomach ; the bowels loose, and often bloody stools. The fatty constituents are removed, and the patient becomes emaciated ; no part of the body is more affected by mercury than the nervous system ; the body trembles ; sometimes one limb, and again both limbs contract. A sense of coldness and occasional chills are experienced ; often neuralgic pains are felt, particularly around the motor nerves ; mental debility and loss of memory. These are some of the many symptoms caused by the inhalation of the vapor of mercury.—*E. S. Talbot, M.D., D.D.S., in Ohio State Journal of Dental Science.*

